# The Work of Incurable Patients

**Published:** 1913-06-28

**Authors:** 


### THE WORK OF INCURABLE PATIENTS.

### Inmates' Sale of Work at the Royal Hospital, Putney.

On Tuesday, Wednesday, and Thursday, last week, the annual sale of inmates' work was held at the Royal Hospital for Incurables at Putney. In its aim, in the objects exposed on the stalls, and in its prices, this sale differs profoundly from charitable bazaars of every kind, and when these characteristics are understood it becomes clear why such a sale of work should be attended and nobly supported by Society and others with more enthusiasm and energy than the bazaar of fashion. The proceeds of the sale go to the patients themselves, and its object is to provide them with pocket-money for the purchase of little delicacies and other personal comforts which the hospital itself cannot provide. When we think, in spite of the cheerfulness which always seems to emanate from those who suffer from an incurable disease, what their lives, at reflective or less happy moments, must be, the object of such a sale as this is seen to appeal to our sympathies more than those functions the proceeds of which less directly and personally benefit the patients.

But there is a more cogent reason why at this sale every object makes a special demand on the visitor. The articles on all the stalls except three have been made by the patients themselves, and even the simplest piece of knitting or embroidery, the apparently most facile decoration of a box or tray, may have been the result of months of interrupted labour, and may represent a few intervals from pain in many weeks or even months of illness. Few things are more startling than the aptitudes which may be cultivated under difficulties, and, apart from any considerations of personal handicap in the makers of the articles on view, the general standard of workmanship is surprising. The knitted shawls, the embroidered drapery, the carved boxes, the dressed dolls, have almost invariably one quality in common—that, namely, of achieving the effect aimed at, whether a constrained choice has made it easy or difficult. The articles are priced by the patients and numbered, and the prices charged on the objects which we made a point of inspecting were not charity figures, but commercial ones. An embroidered tea-cosy of the design and material seen here would not be obtainable cheaper at any of the London shops. The patients know that they must charge for the result which they have been able to gain, and not for the labour involved in gaining it. Were a charge for their labour hoped for, it would be difficult to over-estimate the price.

There are, however, three stalls, the articles on which have been given by friends, and the proceeds of these are divided among those patients who are incapable of making anything. What that must mean comes home to the observer who remembers how much skill is shown by those incurables who still are capable of work. The moral of the sale at first seems to be that there is no such thing as incapacity; but the three stalls for non-workers are witnesses that for some this is indeed complete. Those also whose work chance has left unsold, or whose return from the sale is very slender, also benefit from the proceeds of the non-workers' stalls. Certain ill-fated objects, as the friends of the patients know, return more than once to their maker, and are kept to be sent up again next year in the hope one day to be sold. That, the worst of disappointments, is somewhat mitigated by the sales of the non-workers' stalls.

Such a sale of work in any case demands great sympathy and help from the immediate supporters of the institution, and the majority of the stall-holders are friends of the patients, just as the people in the neighbourhood always appear to buy. But there is no reason, except want of publicity, why a far larger circle should not attend. To what other bazaar can we go knowing that every object will have a real *cachet* about it? By what other bazaar can so much be done, first, to increase the personal comforts of the patients, and, secondly, to make life worth living to them by showing how every spark of their vitality is appreciated. For the articles made by incurable patients do represent precisely what sparks of vitality are left to them; and the man or woman who purchases the things which they have made is assuredly fanning that spark by rewarding its produce. The honorary secretary of the sale of work is Mrs. Casher, who has been its active organiser for very many years, and the enthusiasm with which this voluntary work has filled her is a reflex of the interest with which the making of these things fills the lives of the patients. The incurables become, by its means, producers once more, and, having shown what they can do, the public should nobly support them. If the sale is a success every patient will feel that he or she is still of use in the world. As the bazaar is unique in its object it should be unique in its result. The public ought to make it a point of honour that every article be sold.

